# Nanomaterials to address the genesis of antibiotic resistance in *Escherichia coli*

**DOI:** 10.3389/fcimb.2022.946184

**Published:** 2023-01-04

**Authors:** Mahima Kaushik, Niloy Sarkar, Amit Singh, Pankaj Kumar

**Affiliations:** ^1^ Nano-Bioconjugate Chemistry Lab, Cluster Innovation Centre, University of Delhi, Delhi, India; ^2^ Department of Environmental Studies, University of Delhi, Delhi, India; ^3^ Department of Chemistry, University of Delhi, Delhi, India

**Keywords:** *Escherichia* pollution, microbial pollution, nanomaterial for mitigation, nano-intervention, antimicrobial nanoparticles, *E. coli* pollutants, antibiotic resistance, superbugs

## Abstract

*Escherichia* is a genus of prokaryotic gram-negative bacteria which forms a vital component of the gut microbiota of homeotherms including humans. Many members of this genus are commensals and pathogenic strains, which are responsible for some of the most common bacterial infections and can be fatal, particularly in the case of newborns and children. The fecal matter in wastewater treatment plants serves as major environmental sinks for the accumulation of *Escherichia*. The rise in antibiotic pollution and the lateral gene exchange of antibiotic-resistant genes have created antibiotic-resistant *Escherichia* strains that are often called superbugs. Antibiotic resistance has reached a crisis level that nowadays existing antibiotics are no longer effective. One way of tackling this emerging concern is by using nanomaterials. Punitively, nanomaterials can be used by conjugating with antibodies, biomolecules, and peptides to reduce antibiotic usage, whereas, preventatively, they can be used as either nano-antimicrobial additives or nano-photocatalytic sheets to reduce the microbial population and target the superbugs of environmental *Escherichia*. In this review, we have explored the threat posed by pathogenic *Escherichia* strains in the environment, especially in the context of antibiotic-resistant strains. Along with this, we have discussed some nanomaterial-mediated strategies in which the problem can be addressed by using nanomaterials as nanophotocatalytics, antimicrobial additives, drugs, and drug conjugates. This review also presents a brief overview of the ecological threats posed by the overuse of nanomaterials which warrants a balanced and judicious approach to the problem.

## 1 Introduction

Microbial pollution is the result of the increasing human population coupled with urbanization and animal husbandry. Among microbial pollutants, one of the primary representatives are from the genus *Escherichia* which are gram-negative, non-spore-forming, facultatively anaerobic, and rod-shaped bacteria. The species of the genus *Escherichia* are almost universal inhabitants of the gastrointestinal (GI) tract of homeotherms including humans, but unlike other genera of the same family such as *Salmonella* or *Shigella*, they are mostly non-pathogenic (Brock et al., 2003). The problem of microbial pollutants has been compounded by antibiotic micropollutants, which are also a by-product of the increasing population and originate both as effluents of pharmaceutical plants and cleared through the GI tract. Since the mobility of both gastrointestinal microbes, including opportunistic pathogens such as *Escherichia coli* and excreted micropollutants such as antibiotic residues, is waterborne and dictated by the water cycle, the solution must be at the point of concentration (sink) of both the microbe and antibiotic, which in this case are wastewater treatment plants. The anthropogenic concentration of *Escherichia* micropollutants and the proximity with antibiotic residues lead to the emergence of pathogenesis and antibiotic resistance which are a matter of great concern. In this review, we have focused on *E. coli*, which is one of the best-studied and characterized model organisms and a representative of its genus (Yu et al, 2021). In most cases, *E. coli* exists as a harmless commensal but can develop pathogenesis and lead to bacterium-related diarrhea, urinary tract infections, and meningitis (Palaniappan et al., 2006). The study of *E. coli* is also important as it is one of the bacteria which poses the greatest risk to human health due to its growing resistance to antibiotics and has been classified by the World Health Organization (WHO) along with the rest of the *Enterobacteriaceae* family as the 12 families of bacteria which pose the greatest threat to human health (Galindo-Méndez, 2020). Predictive statistical modeling of 23 pathogens has been used to estimate the global total deaths associated with and attributable to antimicrobial resistance of bacteria in 2019 to be 4.95 million and 1.27 million, respectively, with *E. coli* being responsible for most of the deaths with those associated with and attributable to antimicrobial resistant *E. coli* at 23.4% and 24.3%, respectively (Fernandez, 2022). Even without antimicrobial resistance, *E. coli* poses a significant health risk, as it is one of the major bacterial species contributing to diarrheal diseases and remains the second biggest killer of children below 5 years old after pneumonia, causing 0.437 million deaths annually (International Vaccine Access Center, 2020; Karambizi et al., 2021).

We have discussed the ecological role and changes of *E. coli* caused by the increased human population and also touched on the emergence of pathogenesis in the bacterium and how the problem is magnified in its artificial sink, wastewater treatment plants (WWTPs), where pathogenesis may provide survival benefit to the bacteria. In addition to pathogenesis, exposure to sublethal levels of antibiotics exerts a directional evolutionary selective pressure. Given the challenges, the use of nanoparticles (NPs) and associated nanomaterials has emerged as an attractive countermeasure either used in conjunction with antibiotics to reduce their effective dosage by increasing their efficiency or used on their own as nanotoxic additives or nanophotocatalytic sheets to neutralize microbial pollutants in their sink. The usage of nanomaterials is diversified in various sectors such as in industries, cosmetics, protective and self-cleaning materials, and most importantly, medicine. Recently, the use of nanoparticles showed remarkable antimicrobial properties. The properties of nanomaterials help in improving conjugation with antibiotics, providing better release, reducing the required dose, improving solubility, and enhancing better uptake and bioavailability. Novel nanomaterials are useful for both the source and the sink to address the problem. Nanoformulations can be used to increase the efficiency of antibiotic medication and, therefore, reduce their excretory load in wastewater. Also, their unique electro-optical and surface redox properties can help in reducing the micropollutant load in the sink, making them an ideal choice to address this concerning matter.

## 2 The ecological role of *Escherichia coli*

The gut microbiota accounts for 10^10^-10^11^ cells per gram of large intestinal content, consisting of more than 500 different species of bacterium (Tenaillon et al., 2010). Anaerobic bacteria outnumber *E. coli* 10^2^–10^4^ times; however, the latter can survive in environmental sediment, hence is most significant (Tenaillon et al., 2010). *Escherichia coli* is also one of the first microbes which colonize the human gut just after birth (Galindo-Méndez, 2020). The gut microbiota is a plastic ecosystem whose dynamics are determined by early microbial exposure, host genetics, and lifestyle (Fabbiano et al., 2017). The species *E. coli* presents an interesting ecological study due to the transition of the genus across mutualism, commensalism, and pathogenesis (Braz et al., 2020). In the mutualistic role, certain strains of *E. coli* are responsible for the production of vitamin K_2_ (menaquinone) which aids in clotting (Reygaert, 2017). The gut microbiota, including *E. coli*, contributes not only to host nutrition but also to host food metabolism, drug metabolism, and immune system development (Fabbiano et al., 2017). Gut microbiota including *E. coli* produces short-chain fatty acids (SCFA) which have been shown to play a role in the immune system and can reduce inflammation where chronic inflammation has been shown to play a role in colorectal cancer (Nakkarach et al., 2021). Healthy microbiota can also prevent the establishment and expansion of potentially pathogenic microorganisms in a process called colonization resistance either *via* bacteria–bacteria interventions or by triggering the host immune system (Sorbara & Pamer, 2019). Commensal strains of *E. coli* can provide colonization resistance against pathogenic serotypes and other genera such as *Salmonella* by resource competition for sugars present on the mucous layer or trace metals, respectively (Sorbara & Pamer, 2019). In its commensal role, *E. coli* still derives nutrition and protection, providing nothing significant in return. In the third form of interaction, pathogeny, certain strains of *E. coli* can cause serious illness, which may even lead to death.

Until recently, it was thought that *E. coli* is not able to survive and replicate for long outside a host; however, that has been challenged by the recent observation of the bacterium surviving for long periods in soil and sediment in tropical, subtropical, and temperate environments (Jang et al., 2017). As mentioned previously, the primary niche of *E. coli* is the colon of endotherms which includes cattle and humans from where it enters the environment with feces in the form of manure and sewage. Once in the environment, *E. coli* can survive for periods of time depending on a multitude of factors, including bacterial load, strain type, temperature, moisture, pH, oxygen, soil texture, and dissolved nutrients, particularly carbon (Van Elsas et al., 2011; Chekabab et al., 2013). Certain strains of the bacterium can respond in different ways including reducing cell size to increase surface area ratio or adopting a transport system with greater nutrient affinity; therefore, improving nutrient scavenging, morphological changes improving adhesion and colonization, and secretion of toxins as seen in certain pathotypes can also improve environmental survival and therefore be favored by natural selection (Van Elsas et al., 2011; Chekabab et al., 2013). Given that a high *E. coli* load is present in feces, the sewage system and wastewater treatment plants naturally serve as an artificial sink for the bacterium as it caters to highly dense habitations of humans in towns and cities. The sewage system and wastewater treatment plants provide many of the conditions which improve the survival of *E. coli* including an anaerobic environment rich in dissolved nutrients.

**Figure 1:**
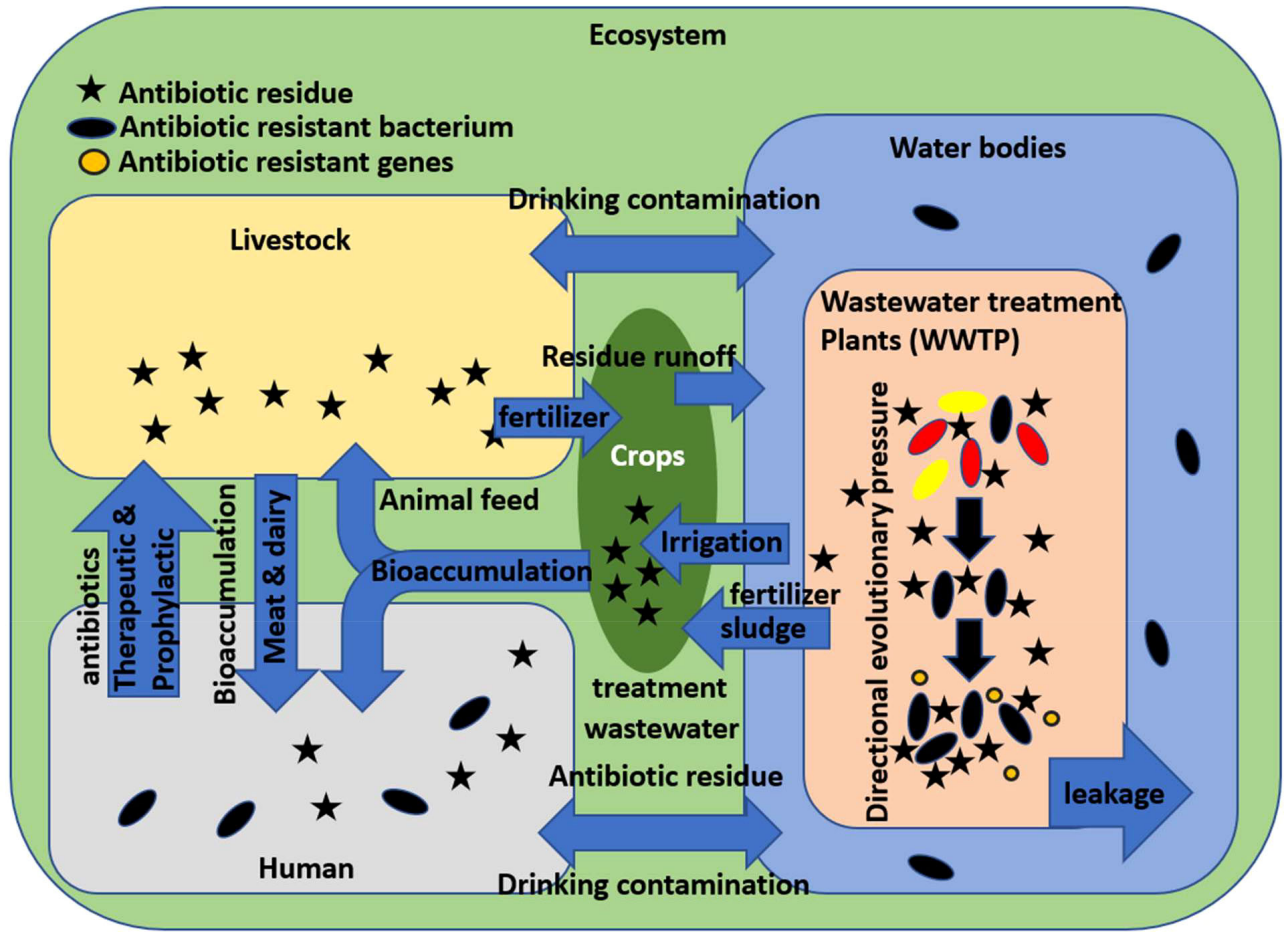
Ecological cycle of antibiotic resistance development in *Escherichia coli* and human exposure.

**Figure 1** is a representation of the environmental cycle of *E. coli* and how it encounters antibiotic residue. As stated earlier, *E. coli* is mostly an intestinal commensal of warm-blooded animals, which includes in this context humans and livestock. Antibiotics are consumed by humans for therapeutic purposes and are administered to livestock for both therapeutic and prophylactic purposes to maximize economic productivity. In both cases, *E. coli* is excreted daily in the form of feces, where it may already be exposed to some antibiotic residues. Large quantities of livestock wastes are used as fertilizers, from where the *E. coli* can enter freshwater *via* surface runoff. On the other hand, human wastes, in the form of sewage, end up in wastewater treatment plants, where wastewater is mainly treated to separate debris and reduce organic matter. In a conventional wastewater treatment plant, the wastewater is first subjected to a grit and sand filter to remove debris. Then, it goes to the primary settling chamber to remove unstable colloidal contaminants. This is followed by an aeration tank to oxidize any organic matter into carbon dioxide and simpler organic compounds. Then, secondary settling is done with microbial additives, which feed on the simpler organic matter and in turn increase in bulk and create activated sludge, which is later reused and recycled. The final step includes disinfection with chlorination, ozonation, or UV radiation exposure, and finally, the cleaned water is released back into natural water. The problem with this method is that while it is good for the treatment of biological organic wastes, it is not purposely directed at treating specific ligands such as antibiotics. Micropollutants are often resistant to degradation due to their closed ring structure, non-biodegradability, and stability. Pharma micropollutants are present in low concentrations but are dangerous due to their persistence and, thus, possible ability to affect an organism’s entire life cycle (Petrovic et al., 2004; Gavrilescu et al., 2015). It is in the settling tanks of wastewater treatment plants where a large population of microbes including *E. coli* is brought in contact with antibiotic residues and organic matter. The microbial population is subjected to both directional evolutionary pressures as well as lateral gene transfer by mobile genetic elements and may develop antibiotic resistance. From here, the microbes and antibiotic residue may enter freshwater either *via* release and ineffective sterilization or through leakage. Freshwater with both microbial and antibiotic contamination is used for irrigation and drinking for both humans and livestock, whereas sludge from a wastewater treatment plant that contains microbial biomass is used as fertilizer on crops. The usage of both contaminated water and sludge for drinking, irrigation, and fertilizer and eating contaminated animal products may lead to bioaccumulation of antibiotic residues as well as exposure to antibiotic-resistant bacteria including *E. coli.* Animal husbandry and agriculture require large amounts of water to produce and process their products. Raw meat and vegetables are susceptible to carry large numbers of bacteria if processed with inadequately treated water (Rasheed et al., 2014). It is important to keep in mind that this is a multidirectional process.

## 3 Pathogenesis in *Escherichia coli* and the role of antibiotics

The path to pathogenesis is rooted in the genome of *E. coli* which contains all the tools that the bacterium uses to adapt and survive in its microhabitat. Genomic evolution is driven by a multitude of internal and external forces including mutation, recombination, horizontal gene transfer (HGT), and directional evolutionary pressures. One reason behind the pathogenesis of *E. coli* is the substantial genetic plasticity of its pan-genome which may be thought of as a mosaic with a set of core and accessory genome, virulence genes, and antibiotic resistance in transmissible genetic elements which can easily be shared among different bacteria (Braz et al., 2020). These pathotypes have diversified their niche beyond the colon and into the small intestine, urinary tract, and meninges leading to diarrheal diseases, urinary tract infection (UTI), meningitis, and sepsis. Pathotypes have several morphological adaptations which aid in colonization and adhesion which help them establish themselves (Lawal et al., 2022). *Escherichia coli* is the most frequent cause of urinary tract infections and has been identified as the causative agent behind the disease in every anatomical site of the body leading to appendicitis, pneumonia, gastrointestinal infections, skin abscesses, endocarditis, meningitis, and intra-amniotic and puerperal infections in pregnant women (Galindo-Méndez, 2020).

**Figure 2:**
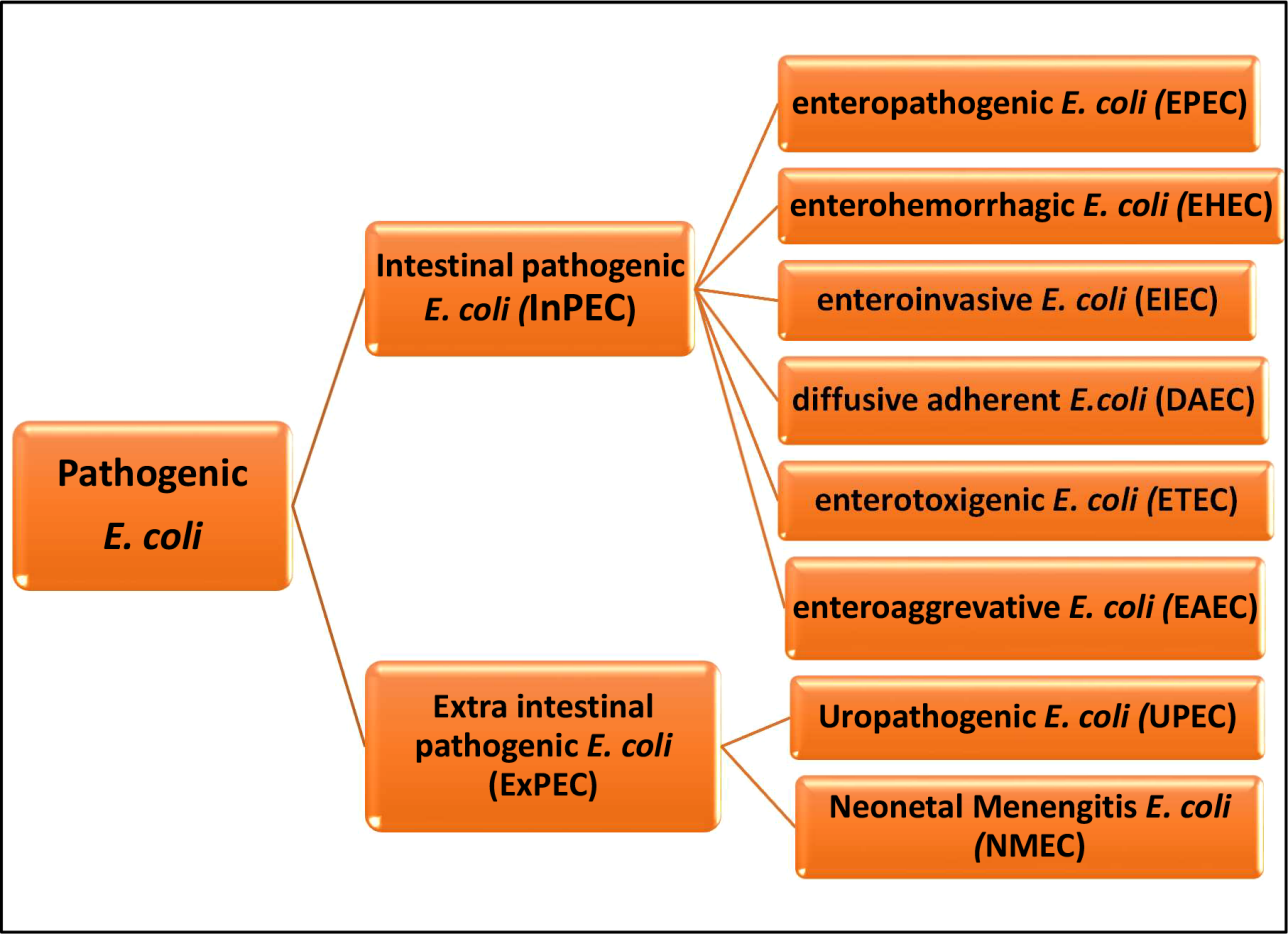
Basic *Escherichia coli* pathotypes.

A basic schematic of *E. coli* pathotypes is given in **Figure 2**. Based on the virulence factors present, pathogenic *E. coli* can be classified into the following: enteropathogenic *E. coli* (EPEC) which causes diarrhea in children and animals; enterohemorrhagic *E. coli* (EHEC) which causes hemorrhagic colitis and hemolytic-uremic syndrome; entero-invasive *E. coli* (EIEC) responsible for inflammatory diarrhea; enterotoxigenic *E. coli* (ETEC) which causes traveler’s, porcine, and bovine diarrhea; entero-aggravative *E. coli* (EAEC) which causes persistent diarrhea; diffusively adherent *E. coli* (DAEC) which is also responsible for diarrhea in children; entero-invasive *E. coli* which causes watery diarrhea and dysentery; uropathogenic *E. coli* (UPEC) which causes urinary tract infections; and neonatal meningitis *E. coli* (NMEC) which is responsible for meningitis and sepsis (Palaniappan et al., 2006; Kang et al., 2013; Rojas-Lopez et al., 2018).

This problem of *E. coli* microbial pollution is compounded by the fact that they are also exposed to antibiotic residues in sewage and wastewater treatment facilities. The consumption of antibiotics has increased to an estimated 14.2 defined daily doses (DDD) per 1,000 per day in 2018, which is a 46% increase from 2000 (Browne et al., 2021). Applying the principle of what goes in must also come out, undigested antibiotics, as well as residue molecules from not only humans but also cattle, are released into the sewage system. Antibiotics apply a directional evolutionary pressure on the *E. coli* microbes and lead to the development of antibiotic resistance genes which along with virulence-causing elements can be exchanged among *E. coli* microbes. The development of pathogenesis and antibiotic resistance poses a compounding risk of antibiotic-resistant pathogenic *E. coli* which poses a serious threat to human health. Antibiotic resistance may be a result of the bacterium’s inherent makeup, acquired traits, or active measures. Recent advances in whole genome sequencing have shed light on the genetic basis of the concern. *Escherichia coli* has a pan-genome of about 18,000 genes, with the average and core genomes of about 4,700 and 2,000 genes, and evolutionary pressures and horizontal gene transfer of genetic elements are the major reasons behind virulence and antibiotic resistance (Van Elsas et al., 2011; Poirel et al., 2018). Active measures which can be used by *E. coli* against drugs include limiting the uptake of the drug, modifying the drug target, drug inactivation, and drug efflux (Reygaert, 2017). One way to overpower bacterial countermeasures and effectively neutralize them before they enter the environment and cause diseases is *via* using nanomaterials. Nanomaterials can be used either as nanophotocatalysts that use solar or artificial radiation to physically lyse the cell or as nanotoxic additives for microbes or as conjugates to antibiotics to reduce their needed amount or improve their efficiency.

## 4 Threats posed by antibiotic-resistant *Escherichia coli*

As previously mentioned, the use of antibiotics has gone up considerably in the past few decades, especially in the developing world, for both human and livestock applications. This translates into a higher concentration of antibiotic residue in wastewater. *Escherichia coli* is a very common commensal organism, which inhabits the gastrointestinal tract of warm-blooded animals including humans and thus gets excreted into the sewage system with feces. The bacterium is subject to directional evolutionary pressure from these antibiotic residues, and using its substantial pan-genome, it is able to mobilize antibiotic resistance phenotypes. Antibiotics and antibiotic resistance are part of the natural arm’s race of the microbial world, which has been ongoing for millions of years; thus, microbes have developed antibiotic resistance, and new types of antibiotics have been developed to overcome them. Generally, the cytological and biochemical mechanism of antibiotic resistance can be divided into the following: antibiotic modification or degradation using special enzymes, antibiotic efflux using efflux pumps, and antibiotic sequestration using antibiotic binding proteins, target modification, bypass, or protection which includes strategies from methylation of the target macromolecule to increasing the production of macromolecule variants, which have much less binding affinity to the antibiotics (Peterson et al., 2018). In the case of gram-negative bacterium, two major contributors to intrinsic resistance are the outer membrane and the presence of numerous efflux pumps, which not only prevent the entry of many molecules but also rapidly reduce their intercellular concentration (Galindo-Méndez, 2020).

Data show that *E. coli* presents antibiotic resistance against drugs, which have been used for the longest time, as evidenced by the high degree of resistance against sulfonamides, which have been used globally since the 1930s, and resistant clones were first isolated as early as the 1950s (Galindo-Méndez, 2020). It is also not a coincidence that low- to mid-income countries, which have the highest consumption rates of antibiotics, have a higher rate of antibiotic resistance than high-income countries (Galindo-Méndez, 2020). Over the past decades, *E. coli* has resistance to major classes of antibiotics such as β-lactams, quinolones, aminoglycosides, sulfonamides, and fosfomycin. Unfortunately, this resistance has spread to the last resources of antibiotic classes such as polymyxins and carbapenems (Galindo-Méndez, 2020).

## 5 Nanophotocatalytic sheets for reduction in *Escherichia coli* load

Nanomaterials lie in a transitional realm between bulk and quantum phases of matter and are therefore endowed with special properties, one of them being photocatalysm. In a bulk crystalline conductor, the charge carriers are free flowing; thus, the electronic properties are indistinct. However, in the case where the size of the crystalline material decreases and reaches below the De Broglie electron wavelength, the charge-carrying electrons become confined, leading to unique properties (Daniel & Astruc, 2004; Neikov, 2009). One of these unique properties is called the quantum size effect (QSE) which arises due to the previously mentioned confinement of electrons which causes the edges of the valence band (VB) and conduction band (CB) to split, separated by an energy or band gap (E_g_). The CB is electron-filled and of lower energy, whereas the VB is electron empty and of higher energy (Ajiboye et al., 2021). When a photon of sufficient energy equal to or greater than the band gap (E_g_ ≤ hv) is absorbed by the nanomaterial, electron(s) can be promoted from a lower energy valence band to a higher energy conduction band, leaving behind electron holes (Ajiboye et al., 2021). The exciton pair, electron, and electron hole can cause redox reactions either directly or *via* the formation of intermediate reactive oxygen species (ROS). Various nanomaterials include noble metals (Ag, Au, Pd), metal oxides, metal halides, metal sulfides, metal phosphides, non-metallic nanoparticles such as 3D-reduced graphene oxide (R-GO), doped metal nanomaterials by either other metals or non-metals, and heterojunction nanomaterials which combine wide and narrow band materials (Ajiboye et al., 2021). In addition to nanophotocatalysis, the nanomaterials themselves may show antimicrobial activity due to a multitude of reasons. The exact mechanism behind antimicrobial action is known but has been theorized to include ROS generation, metal ion leaching which can inhibit various biochemical and cytological processes, membrane destabilization, and altering of the cellular permeability (Rajak et al., 2020). Mechanical abrasion and damage of the cell membrane may be another mechanism of antimicrobial activity.

There are three possible ways theorized for the nano-inactivation of bacteria: DNA destruction, oxidative damage of the respiratory enzyme coenzyme A, and damage to the cell wall leading to cytolysis (Ajiboye et al., 2021). In the context of nanophotocatalysis when we are considering nanophotocatalytic sheets and surface reactions, the mechanism of cell membrane destruction is most relevant. The cell wall of *E. coli* is made up of a thick layer of proteins and sugar which prevents cytolysis (Ajiboye et al., 2021). *Escherichia coli* is a gram-negative bacterium which means that its cell wall consists of two lipid membranes with an intermediate peptidoglycan layer.

**Figure 3:**
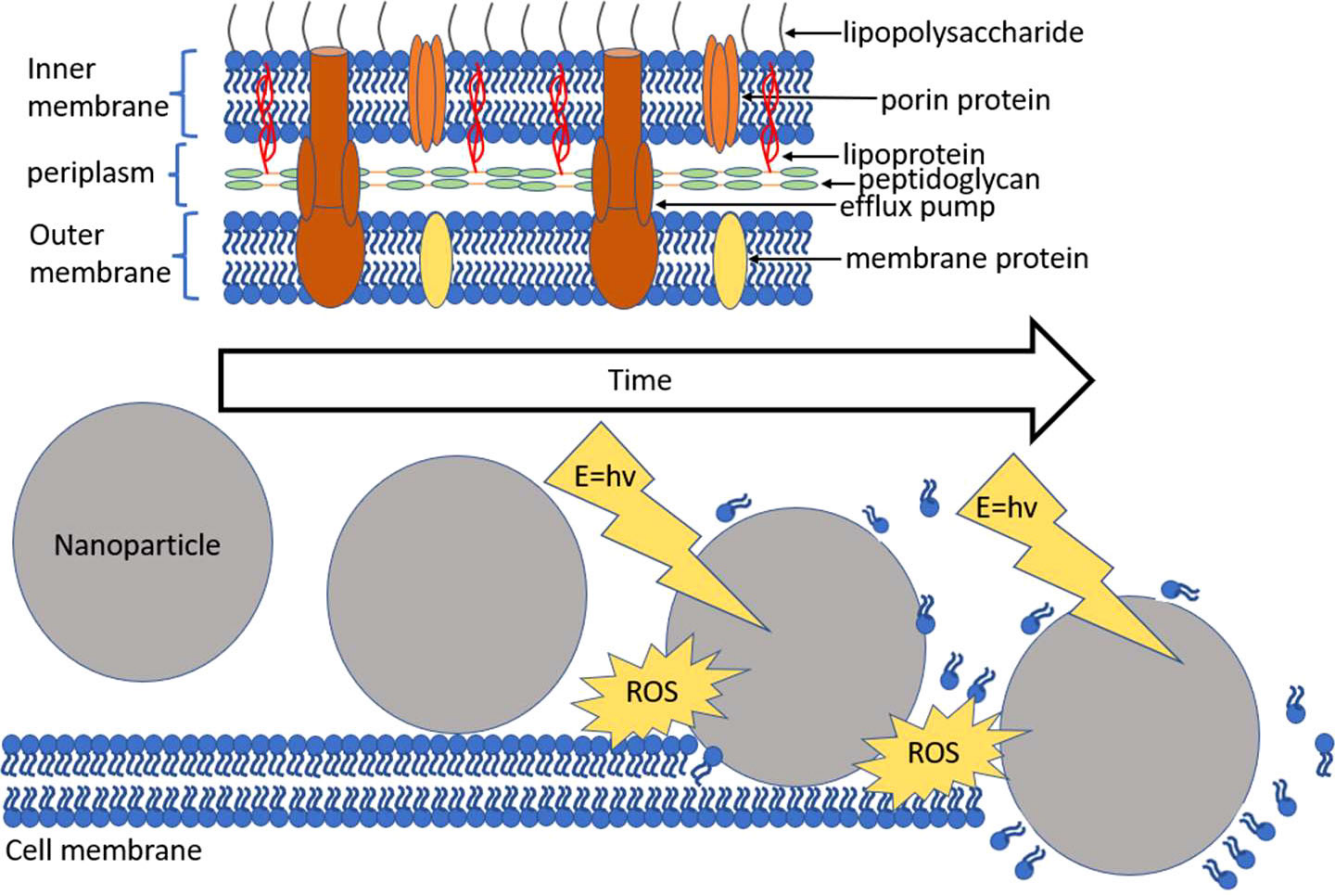
Gram-negative cell membrane with a nanophotocatalytic mode of destruction.

Damage to the bacterial cell wall may be further subdivided into mechanical damage, involving abrasion of the cell onto the nanostructures leading to physical ruptures and photocatalytic damage which depends on the spectra and intensity of incident radiation, concentration, size, morphology, and constitution of the nanophotocatalytic material. The characteristics of an ideal nanophotocatalyst are as follows: it is non-toxic by itself, is chemically and ecologically inert, and has a moderate band gap. Such nanomaterials can either be added in wastewater treatment plants or immobilized on a substratum and exposed to sunlight or artificial light. **Figure 3** provides a representation of both a simplified gram-negative cell wall as well as the photocatalytic mode of destabilization of the cell membrane where minimal damage is done by contact alone if the nanomaterial has a smooth surface, and it is only on incident radiation and ROS generation that significant cell membrane destabilization and delamination occur.

**Table 1** provides a summary of some nanophotocatalytic materials used for *E. coli* neutralization. Immobilization of nanomaterials onto substrates to form nanophotocatalytic screens can be broadly classified as non-covalent and covalent techniques. Non-covalent immobilization mainly involves the electrostatic attraction and adherence of nanomaterials onto a substratum by virtue of opposition of their surface charge. Nano-electrodeposition is a type of electrodeposition involving nanomaterials. The process has many advantages such as direct attachment or growth of nanomaterials on the substratum without a linker and more control over particle size, crystalline orientation, film thickness, and morphology (Mohanty, 2011).

**Table 1:** Nanophotocatalysts used to disinfect *Escherichia coli* contamination from water.

S. no.	Nanomaterial used	Illumination	*E. coli* and NP conc.	Remarks	Reference
1.	TiO_2_ coated on cellulose substratum 78.54 cm^2^	UV-LED at 365 nm and dose of 57,200 mJ/cm^2^	10^3^ and 10^4^ CFU/ml for distilled water (DW) and 5 mg/L of humic acid (HA), respectively, in a closed loop of 50 ml	Up to 100% inactivation in DW and HA water in 1 and 2 h, respectively	De Vietro et al. (2019)
2.	Fe-doped ZnO (Fe-ZnO) NPs 0.25 g/L	Sunlight with 100,000 ± 5,000 lux	1.2 × 10^7^ CFU/ml in 500 ml closed loop in deionized water	Complete disinfection of multiple drug-resistant *E. coli* in 1.5 h	Das et al. (2017)
3.	TiO_2_ coating on stainless steel 96 cm^2^	UVA radiation at 365 nm at 3 mV/cm^2^	Experimental setup as per standard BS EN 13697:2001	Complete disinfection in 2-3 h	Evans & Sheel (2007)
4.	TiO_2_/SiO_2_ 0.05 g/L	UVA lamp at 365 nm at 125 W	10^6^ CFU/ml in 1-L reaction flask in Milli-Q water with organic additives	1 h complete disinfection	Marugan et al. (2009)
5.	Ag-TiO_2_ 0.75 g/L	Mercury pressure lamp 250 W	10^7^ CFU/ml solution of 25 ml under constant agitation	16 m complete disinfection	Reddy et al. (2007)
6.	Ag-ZnO 0.25 g/L	Solar light	1.1 × 10^9^ CFU/ml solution in constantly agitated batch	1 h complete disinfection	Adhikari et al. (2015)
7.	AgBr/AgVO_3_ 0.4 g/L	800 W Xe lamp	3.54 × 10^7^ CFU/ml in sterilized seawater	0.5 h for near-complete disinfection	Zhang et al. (2019)
8.	Ag/TiO_2_/ZnO 0.4 g/L	Mercury vapor lamp	10^7^ CFU/ml in 50 ml agitation beaker	1.3 h for near complete disinfection	Pant et al. (2013)
9.	ZnO 0.4 g/L	Mercury vapor lamp	10^7^ CFU/ml in 50 ml agitation beaker	2 h for near complete disinfection	Pant et al. (2013)
10.	Conjugated polymer at CuO 1.3 g/L	Xenon lamp 300-1,100 nm	10^7^ CFU/ml stock in 15 ml LB broth	1.5 h for near complete disinfection	Djellabi et al. (2020)
11.	Ag-Fe_3_O_4_/graphene-C_3_N_4_ nanosheets 0.4 g/L	Sunlight	1.5 × 10^7^ CFU/ml in LB broth constant agitation	2 h for near complete disinfection	Pant et al. (2017)
12.	TiO_2_-reduced graphene oxide 0.5 g/L	Sunlight	10^6^ CFU/ml in isotonic solution agitated	0.3 h for near complete disinfection	Fernandez-Ibanez et al. (2015)

An electrodeposition setup consists of the following components: an anode which may be the nanomaterial precursor or inert, a cathode which is the working electrode or where deposition is to be done, an electrolytic bath or solution, a voltage source, and a power management device. On application of a potential difference, electrons are replaced from the anode to the cathode, nanomaterial precursor ligand cations migrate to the cathode under the electric field, but reaction with a reductant present in the solution can cause agglomeration into nascent nanomaterial clusters, followed by growth and/or immobilization on the cathode (Cheon et al., 2011). Electrodeposition properties such as electrolytic bath constituents and concentration, working electrode surface area, temperature, time, current/voltage, and whether it is continuous or pulsed can be altered to fine-tune the morphology and size of deposited nanomaterials. Nanocomposites can be created depending on the electrodeposition in the presence of several nanomaterial precursor chemicals. If the nanomaterial has been prepared beforehand, then using the same technique can be electrodeposited on the substratum also.

Physical deposition techniques are cheaper and facile and employ dip or spin coating whereby the nanomaterial is suspended in a suitable solvent and the substratum is either dipped into the solvent or fixed on a rotating platform and a drop of solvent is placed followed by spinning. This results in a thin film of solvent and nanomaterials to be deposited on the substratum, and the film thickness can be controlled by the nanomaterial concentration in the solution and RPM. Vapor deposition is another technique that can be used for fabricating controlled nanolayers on a substratum. Vapor deposition can be further subdivided into chemical vapor deposition (CVD) and physical vapor deposition (PVD). In chemical vapor deposition, the nanomaterial precursor chemical is present in the gaseous phase (vapor) and is activated *via* heat, light, and plasma and then exposed to the substratum where it reacts to form stable solid nanostructures (Choy, 2003). PVD is performed in a high-vacuum environment where the target or precursor nanomaterial is present in a condensed form and is bombarded by a sputtering gas, detached atoms, under the influence of an electrical field deposit on the substratum (Safavi et al., 2021). An additional step of calcination can also be employed where the substratum with an overlaying thin film is subjected to high temperature for several hours which results in the evaporation of the solvent, any volatile impurities, and adhesion between the nanomaterial and underlying substratum.

Covalent immobilization techniques involve the covalent chemical linking of nanoparticles onto the substrate. The benefit of this method is that the nanomaterial can be synthesized and stored separately, and covalent bonds are strong, hence resistant to detachment on extreme changes in temperature, pH, electrical fields, and mechanical abrasion. Several crosslinking chemical reactions including NHS-EDC and biotinylation can be used. NHS-EDC crosslinking involves the formation of a stable NHS ester intermediate and ultimately links the carboxylic group with amine. Biotinylation is another crosslinking process involving the linking of biotin with another molecule such as streptavidin which binds it with high affinity. The requirement of such techniques is that the prerequisite chemical groups must be present on the surface of the nanomaterial and substratum. If not, then the nanomaterial or substratum can be coated with a suitable ligand to make those groups available for conjugation. The benefit of using nanophotocatalytic sheets instead of the addition of nanomaterials into wastewater directly is that it would limit nanomaterial discharge into the wastewater which would eventually end up in the hydrosphere and ecosystem.

## 6 Role of antimicrobial nanoparticles in mitigating *Escherichia coli* pollution

In the turbid conditions of wastewater treatment plants, nanophotocatalysts reduce in efficiency because of lower availability of light. In such cases, another property of nanomaterials, that is nanotoxicity or the antimicrobial nature of certain nanoformulations, can be used to address the issue. Certain properties of nanoparticles already prove its competency in various fields like drug delivery and sensing. The cell wall of the bacteria provides strength, stiffness, and structure to the cell, while it also helps against osmotic rupture and mechanical damage. The cell wall of any bacteria plays an important role in the diffusion of the nanoparticles in the cytoplasm of the cell. Several other factors can alter the bacteria’s susceptibility or tolerance toward nanoparticles such as pH, concentration, shape, size, charge, and chemical property. Copper oxide (CuO) NPs are extremely sensitive to *E. coli*, but *Staphylococcus aureus* and *Bacillus subtilis* are less susceptible. In another case, *B. subtilis* and *S. aureus* are more sensitive to ZnO NPs and NiO NPs than *E. coli* (Baek & An, 2011).

**Figure 4:**
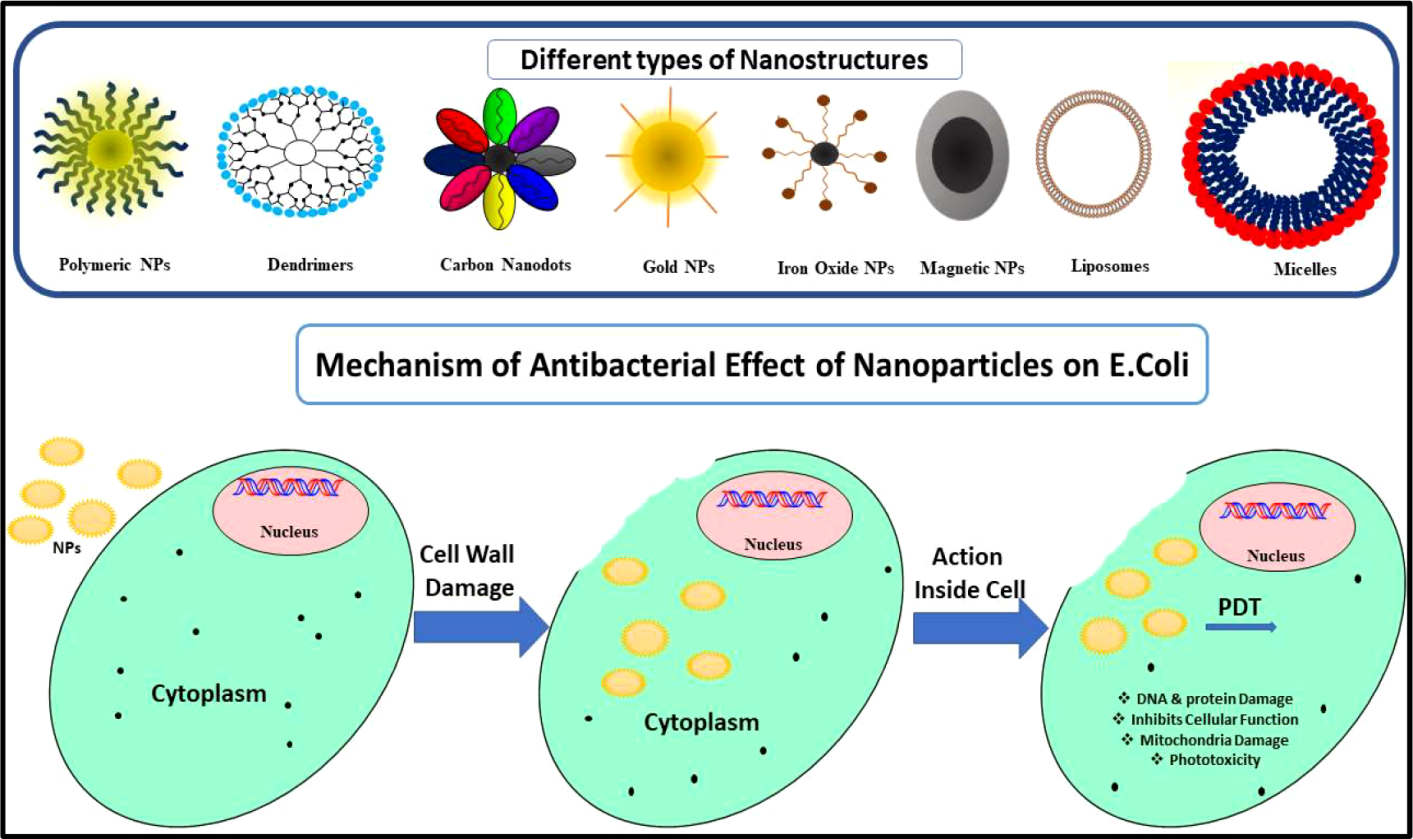
Schematic representation of the toxicology effect of nanoparticles on the bacterial cell.

The mechanism of action and cellular response due to nanoparticle treatment are still areas of exploration. Nanoparticles adhere or attach to the cell membrane of the bacteria by electrostatic attraction and disrupt the membrane integrity which is shown in **Figure 4**. The nanoparticles and bacteria can interact directly by physical contact with the cell membrane or by inducing the irregular pits on the cell wall for the entry of ions in the cytoplasm. It was reported by Vazquez Munoz et al. that nanoparticle interaction with the cell wall can alter the potential of the cell membrane which enhances its permeability for foreign particles (Vazquez-Muñoz et al., 2019). The metallic nanoparticles destabilize the plasma membrane potential and lead to outer membrane destabilization and ATP depletion (Lok et al., 2006).

The properties of nanoparticles such as shape, size, and charge affect the tendency of transfection. Small-size nanoparticles have a higher internalization tendency in bacterial cells, and due to the high surface area to mass ratio, it can affect the bacterial function much predominantly as compared with larger nanoparticles. Other than the size of nanoparticles, shape also plays a vital role in interaction with cells. Among the other shapes of nanoparticles such as rod, sphere, tube, cube, plate, sheet, star, and triangle, the spherical ones are the most used shapes (Slavin et al., 2017). According to the study by Wang et al., the rod- and cube-shaped nanoparticles are more effective due to their high number of crystal facets (Zhipeng Wang et al., 2010). The higher facets with the atom density, the less energy it takes to produce oxygen vacancies, and the stronger the antibacterial action will be. In addition to this, the wire- and rod-shaped nanoparticles show high penetration power in bacterial cell wall as compared with the spherical ones. Talebian et al. reported a greater antibacterial tendency of the flower-shaped nanoparticles as compared with the spherical nanoparticles (Talebian et al., 2013). The charge of the nanoparticles is another factor that affects the transfection of nanoparticles inside the bacterial cell wall. The bacterial cell wall is mostly negatively charged, and the positively charged nanoparticles adhered securely on the bacterial surface before fusing with the cell wall, whereas the negatively charged nanoparticles show no attachment (Ivask et al., 2014). The cationic nanoparticles induce cell membrane damage by penetrating inside it and interfere with and alter the function of the electron transport system of the bacterial cell (Slavin et al., 2017). However, some studies reveal that surface modification of nanoparticles *via* using biocompatible polyethylene glycol, chitosan, polyethylene imine, and 3-aminopropyltriethoxysilane enhances cellular transfection and reduces the chance of rejection (Tamara et al., 2018).

## 7 Possible mechanisms of antibacterial nanoparticles

**Figure 5:**
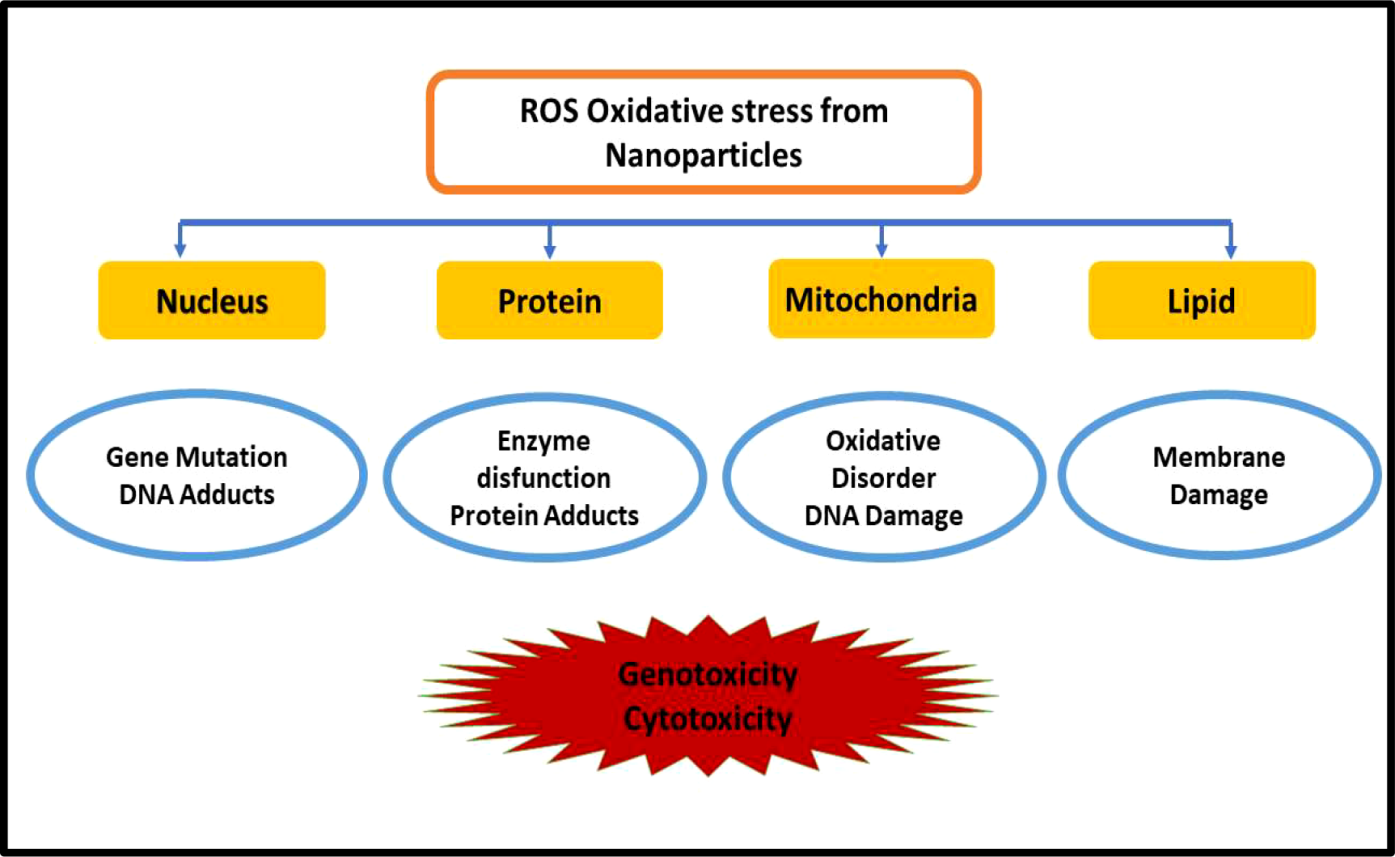
Toxicity induced by nanomaterials by ROS generation.

Several nanosystems showed antimicrobial tendency without the involvement of antimicrobial drugs. Nanosystems show antibacterial property through membrane disruption, ATP depletion, ROS generation, and DNA replication inhibition (Slavin et al., 2017; Tamara et al., 2018). Among various nanomaterials, the metal-based chitosan nanoparticles and surfactant-modified nanoparticles are used frequently for antibacterial action. After the uptake of nanoparticles, these internalized nanoparticles cause nanotoxicity by ROS production which results in the formation of oxidative stress, and it affects various cellular functions which are shown in **Figure 5**.

**Figure 6:**
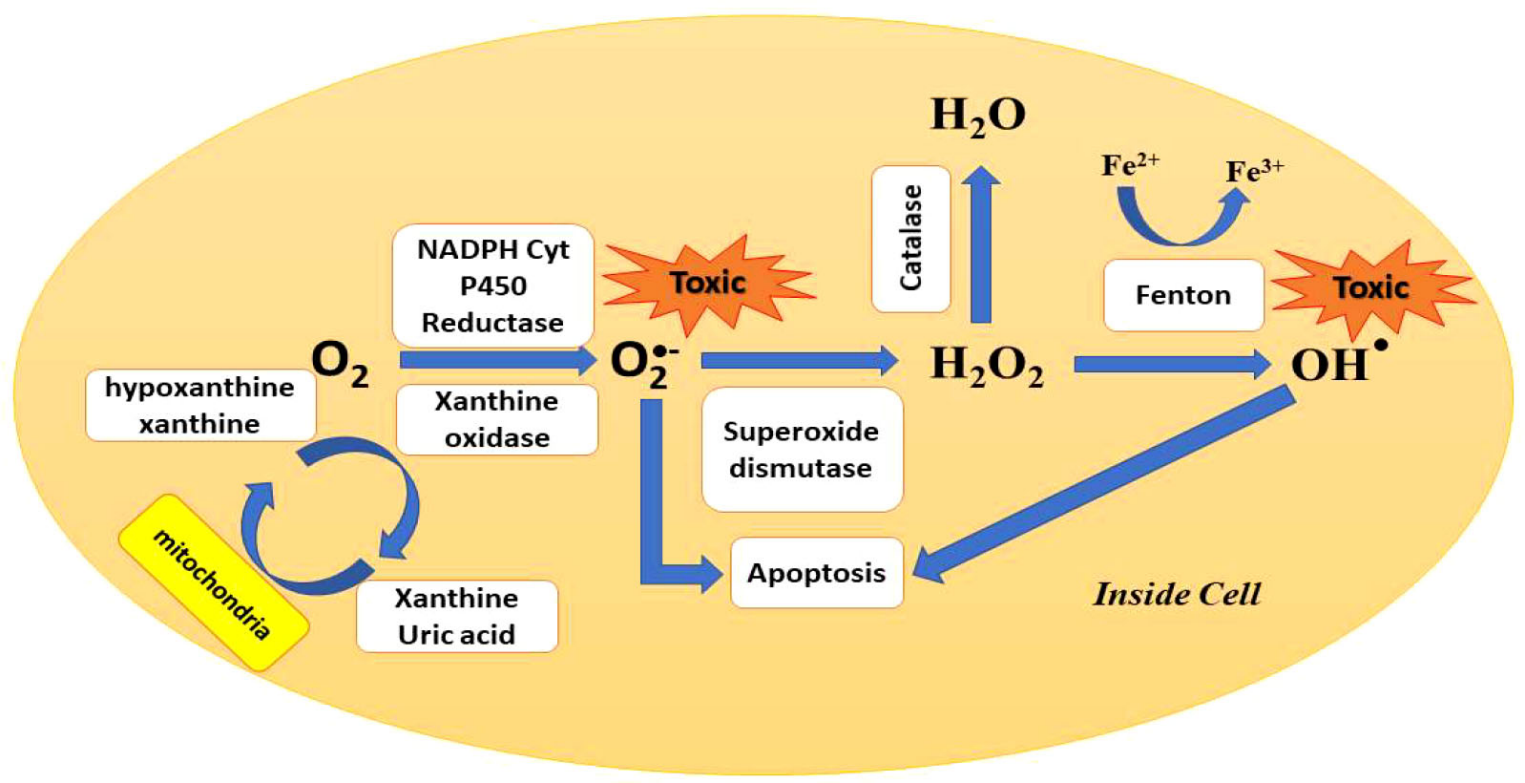
Pathways of ROS production and clearance inside the cell.

The mitochondria of the cell produce ATP by the reduction of molecular oxygen to water *via* a series of proton and electron transfer reactions as described in **Figure 6**. Sometimes, the oxygen is not completely reduced and forms harmful radicals such as superoxide, hydroxyl, hydrogen peroxide, and other oxygen radicals. The ROS plays a beneficial role in mitogenic responses and cellular signaling, but in the case of overproduction of ROS, it is hard for the cell to maintain normal physiological redox-based functions. It has been found from various studies that oxidative stress was the key reason in the case of inflammation, Parkinson’s disease, cardiovascular disease, Alzheimer’s disease, arthritis, amyotrophic lateral sclerosis, diabetes, and cancer (Halliwell, 1989; Bodamyali et al., 2000; Sachidanandam et al., 2005; Valko et al., 2007; Yin et al., 2009). The excessive ROS production by the nanoparticles inside the bacterial cytoplasm causes genotoxicity; oxidative DNA, lipid, and protein damage; mutagenesis; and carcinogenesis in humans (Abo-zeid and Williams, 2020).

The nanotoxicity caused by the overproduction of ROS and the extent of nanotoxicity caused by nanoparticles depend on its chemical nature, structure, shape, and size (Gonzalez et al., 2008). Compared with their bulk counterparts, nanoparticles have much higher surface area and reactivity which lead to high ROS generation leading to genotoxicity and cytotoxicity (Oberdörster et al., 2005). In a study, ZnO nanoparticles easily absorb UV light and produce hydrogen peroxide and superoxide anion, and in the case of TiO_2_, produce radicals after UV irradiation. The produced reactive species can easily diffuse inside the cell and cause cell death (Brunet et al., 2009; Sirelkhatim et al., 2015). Various nanoparticles used against *E. coli* are summarized in **Table 2**.

**Table 2:** Different nanostructured materials and their toxic effects in *Escherichia coli* bacteria.

S. no.	Nanoparticles	Size	Dosage	Mechanism of action	Reference
1.	Ag NPs	5-40 nm	30 μg/ml	Nanoformulations cause cell lysis and prevent DNA unwinding	Fayaz et al. (2010)
2.	Silica NPs	80–100 nm	8 mg/ml	NO-releasing NPs cause biofilm killing of microbes due to electrostatic properties	Hetrick et al. (2009)
3.	Al_2_O_3_ NPs	50-70 nm	20 mg/L	Cause damage to the cell wall	Jiang et al. (2009)
4.	Silica-supported Ag NPs	100-130 nm	1.00 mg/ml	NPs penetrate the cell wall and restrict DNA replication	Gankhuyag et al. (2021)
5.	SiO_2_ NPs	10-20 nm	20 mg/L	Cause damage to the cell wall	Jiang et al. (2009)
6.	Chitosan-coated ZnO NPs	10-30 nm	≥0.0125% of 2% of NPs	Induction of oxidative stress due to ROS	Al-Nabulsi et al. (2020)
7.	ZnO NPs	20 nm	20 mg/L	Damage the cell wall	Jiang et al. (2009)
8.	Fe_2_O_3_	110 nm	10 mM	Elevate the amount of ROS	Li et al. (2018)
9.	NiO NPs	20-30 nm	20 mg/L	Damage cellular functions and structure integrity and inhibit growth significantly	Wang et al. (2010)
10.	Chitosan/protamine hybrid nanoparticles	80-100 nm	31.25 μg/ml	Inhibit the virulence of pathogens by induction of c-di GMP	Tamara et al. (2018)
11.	Cu NPs	25 nm	500 ppm	Generate hydroxyl radical inside the cell	Rispoli et al. (2010)
12.	Fe_2_O_3_ NPs	10-15 nm	250 μg/ml	Inhibit growth by the generation of ROS, superoxide radicals, hydroxide radical, singlet oxygen, hydrogen peroxide	Gabrielyan et al. (2019)
13.	TiO_2_ NPs	20 nm	10 mg/L	TiO_2_ activity is enhanced by photoactivation. Peroxidation of the polyunsaturated phospholipid membrane and loss of respiratory function	Tsuang et al. (2008)
14.	Ag NPs	10 nm	25-100 mg/L	Disturb the permeability of the cell membrane and affect respiration and the cell division process	Morones et al. (2005)
15.	Chitosan NPs	10-100 nm	3.5 mg/ml	Restrict the growth and metabolic activity of *E. coli*	Denisova et al. (2022)
16.	TiO_2_ nanoparticles (NPs) modified with poly-amidoamine dendrimer macromolecule (PAMAM, G3)	50 nm	32 μg/ml	Absorption of ammonium groups, damage cell wall, cytoplasmic membranes grafting, stopping bacterial activation	Matboo et al. (2022)
17.	Ag NPs	20 nm	100 μg/ml	Damage bacterial membrane	Alzubaidy (2022)
18.	Au decorated on coral-like BiFeO_3_	110-140 nm	0.035 to 0.024 mg/L	Photocatalytic inactivation of *E. coli*	Lam et al. (2021)

An additional benefit of using nanomaterials as either photocatalytic or toxicity-mediated bacteriostatic or bactericidal agents is that they can destroy microbes, which have already acquired antibiotic resistance and thus post an immediate threat of release and contamination. Microbes are also capable of acquiring resistance against nanomaterials but that would take multiple mutations and would be achieved through reducing membrane permeability for nanomaterials by more efficient efflux pumps (Lee et al., 2019). Therefore, by combining current therapeutics with nanomaterials, the number of mutations for effective resistance is increased, and thus, the chances that it would happen are reduced. A study involving exposure of ampicillin-resistant bacteria to AuNPs functionalized with ampicillin (AuNPs@Amp) showed that it had bactericidal effects (TA and Lu, 2012). This suggested that the nanoparticles and the antibiotics synergistically were able to overcome microbial antibiotic resistance. Nanomaterials are bacteriostatic or bactericidal in contact with microbes *via* photocatalytic or mechanical damage. Since these methods are non-specific, they would require significant membrane or cell wall modification to mitigate. Nanomaterial-mediated membrane damage is capable of destroying even multiple drug-resistant bacteria (B. Das et al., 2017).

## 8 Nanophotocatalytic destruction of antibiotic residues

Until now, we have discussed about the antimicrobial effects of nanomaterials; however, in order to address the issue of development and proliferation of antibiotic-resistant genes (ARGs) and antibiotic-resistant bacteria (ARBs) holistically, the antibiotic residues in wastewater as well as the genetic elements responsible for the lateral transmission of resistance also have to be addressed. As mentioned earlier, antibiotics are water-soluble and non-biodegradable, contributing to their persistence in the wastewater environment. Globally, some of the most used antibiotic classes are penicillins followed by cephalosporins (Klein et al., 2019) with about a quarter of the antibiotics being consumed in S. Asia led by India and about 10th by China (Browne et al., 2021). CdS-TiO_2_ nanoparticles were used.

Some studies using various nanophotocatalytic materials for the degradation of antibiotic residues in water are provided in **Table 3**. The efficiency of mineralization depends on several factors including the nature of the nanophotocatalyst and its concentration, the strength (wattage) and nature of incident radiation which reaches the nanophotocatalyst, and the concentration and type of antibiotic residue. An ideal nanophotocatalyst should have a suitable band gap to be able to use the visible spectra of light irradiated by the sun and not absorbed by the ozone layer. There are four distinct steps involved in nanophotocatalysis: first is the temporary adsorption of the pollutant or, in this case, antibiotic residue onto the nanophotocatalyst surface either due to random collision or electrostatic attraction. This is followed by exciton generation by the nanophotocatalyst, that is the generation of electron and hole pair after absorbing photonic radiation. The next step involves the migration of electron from the valence to the conduction band which is followed by effecting the redox degradation of the antibiotic residue either directly or through ROS intermediates which are formed by splitting of water molecules. The final step involves the desorption of antibiotic residues from the nanophotocatalyst surface. Complete mineralization involves the breaking down of antibiotic residues into carbon dioxide and water.

**Table 3:** Nanophotocatalytic degradation of select antibiotic classes.

S. no.	Nanomaterial	Light source	Nanomaterial conc.	Antibiotic	Antibiotic conc.	Degradation	Reference
1.	CdS-TiO_2_	300 W Xe lamp	0.6 mg/ml	Penicillin	100 mg/L	88% in 120 min	Wang et al. (2021)
2.	TiO_2_	300 W Xe lamp	1 mg/ml	Tetracycline	20 mg/L	Nearly 100% in 20 min	Wang et al. (2017)
3.	TiO_2_@g-C_3_N_4_	300 W Xe lamp	1 mg/ml	Tetracycline	20 mg/L	Nearly 100% in 9 min	Wang et al. (2017)
4.	g-C_3_N_4_	Sunlight	1 mg/ml	Ciprofloxacin	20 mg/L	78% in 60 min	Pattnaik et al. (2019)
5.	Ce^3+^-doped TiO_2_ NPs	UVA lamp	Thin film 4.15 cm^2^	Amoxicillin	15 mg/L	27% in 120 min	Tiwari et al. (2022)
6.	Ce^3+^ doped TiO_2_ NPs	UVA lamp	Thin film 4.15 cm^2^	Tetracycline	15 mg/L	30% in 120 min	Tiwari et al. (2022)
7.	Fe_3_O_4_@g-C_3_N_4_ magnetic mesoporous NPs	500 W halogen lamp with UV cutoff filter	1 mg/ml	Amoxicillin	91.35 mg/L	90% in 120 min	Mirzaei et al. (2019)
8.	CuO-Fe_2_O_3_	UV lamp	1 mg/ml	Tetracycline	20 mg/L	88% in 80 min	Kaushal et al. (2022)
9.	Mn-doped Cu_2_O	Sunlight	1 mg/ml	Amoxicillin	15 mg/L	80% in 160 min	Gaim et al. (2022)
10.	ZnCr_2_O_4_	Tungsten lamp 200 W	1 mg/ml	Cefaclor	5 mg/L	60% in 180 min	Benrighi et al. (2021)
11.	ZnCr_2_O_4_	Tungsten lamp 200W	1 mg/ml	Cefixime	5 mg/L	70% in 180 min	Benrighi et al. (2021)
12.	ZnCr_2_O_4_	Tungsten lamp 200 W	1 mg/ml	Cefuroxime	5 mg/L	80% in 180 min	Benrighi et al. (2021)
13.	7% Ag-CsPbBr/CN	Xe lamp 300 W with UV filter	1 mg/ml	7-Aminocephal-sporanic acid	10 mg/ml	90% in 140 min	Zhao et al. (2019)
14.	ZnBi_2_O_4_	Sunlight	1 mg/ml	Cefixime	10 mg/L	90% in 30 min	Baaloudj et al. (2021)

## 9 Nanophotocatalytic destruction of mobile genetic elements

As discussed previously, antibiotic resistance is developed in microbes including *E. coli* by directional evolution and stored as genetic instructions. These instructions can be exchanged in a process called horizontal gene transfer which uses extracellular naked DNA to transfer resistance genes to microbes which undergo transformation. *Escherichia coli* is not generally thought to undergo transformation naturally; however, under certain conditions where it is subject to stresses, it may become susceptible to transformation, which mainly involves the formation of membrane pores for the uptake of DNA (Hasegawa et al., 2018). DNA is a polyanion which is resistant to many environmental degradative effects by virtue of its strong phosphate backbone; therefore, nanophotocatalytic advanced oxidative processes are attractive in their active degradation.

Some studies of the degradation of genetic elements using nanophotocatalysis are provided in **Table 4**. As in the case of antibiotic degradation, the extent of degradation depends on the nanophotocatalyst used, its band gap and adsorptive capacity, and the incident radiation both in strength and spectral distribution, as well as the nature of the genetic element. It has been reported that the GC ratio is directly proportional to the rate of destruction (Ren et al., 2018). DNA degradation occurs on the phosphate backbone which converts plasmids into ssDNA and further on smaller oligonucleotides. DNA is a polyanion; hence, having a positively charged nanoparticle would increase electrostatic attraction.

**Table 4:** Nanophotocatalytic degradation of genetic elements.

S. no.	Nanomaterial	Nanomaterial conc.	Light source	Genetic material (GM)	Genetic material conc.	Result	Reference
1.	TiO_2_	0.15 µg/µl	UV lamp	Plasmid DNA seq.	9.63 ng/ml	100% in 45 min	Yang and Wang (2008)
2.	rGO-PH-TiO_2_	0.1 µg/µl	Xe lamp	ampC	2.5 log_10_ CE 100 ng^−1^	100% in 180 min	Karaolia et al. (2018)
3.	CdSe-ZnS-TiO_2_	1.33 cm^2^ film	Hg lamp	pUC18	5 µg/ml	100% in 150 s	Shen et al. (2008)
4.	TiO_2_-modified membrane	4.1 cm^2^	UV lamp	tetC plasmid	10^4^ copies/cm^2^	20.66% in 60 min	Ren et al. (2018)
5.	TiO_2_-modified membrane	4.1 cm^2^	UV lamp	Sul2 plasmid	10^4^ copies/cm^2^	99.45% in 60 min	Ren et al. (2018)

## 10 Nanoparticle–antibiotic conjugates

The use of nanophotocatalysts is discussed in external environmental applications as nanophotocatalytic sheets where they would cause the mitigation of ARBs in wastewater. However, nanomaterials can also be used in conjugation with antibiotics to reduce their load on the environment. Nanomaterials alone are a good alternative for mitigating environmental pathogens. However, they lack specificity for microorganisms, thus causing various adverse effects on non-target eukaryotes (Bondarenko et al., 2013; Heim et al., 2015). According to studies in mice and rats, NPs, for example silver NPs, can trigger unexpected alterations in microbes, resulting in dysbiosis (Van Den Brûle et al., 2015; Williams et al., 2015). Therefore, one of the obstacles facing the development and deployment of NP-based treatments is the issue of specificity and the eradication of adverse effects. As compared with antibiotics, the benefit of using NPs is their capacity to negatively influence pathogens through numerous routes at the same time as discussed in the previous section, preventing bacterial resistance from developing (Wang et al., 2017). However, studies have reported for *E. coli* and *Pseudomonas aeruginosa* developing resistance to Ag NPs by producing flagellin, an adhesive protein, which causes NPs to aggregate and to lose their antibacterial effect (Panáček et al., 2018). Overall, bacterial resistance to NPs has not been well investigated, and further research is needed. Despite this, it can be hypothesized that bacterial resistance to NPs is certain because of selective pressure, even if it is not attributable to genetic alterations (Smerkova et al., 2020). A novel method of addressing this issue would be combining the antimicrobial properties of nanomaterials and antibiotics in a synergistic manner.

The rational encapsulation or conjugation of NPs with antibiotics has been one of the potential strategies for minimizing the detrimental effects of NPs and antibiotics on bacterial equilibrium and improving the selectivity of NPs toward specific pathogens. Antibiotics administered as antibiotic–NP conjugate forms at the same concentration as that of the free antibiotics had better inhibitory effects on bacterial growth, which might be explained by the enhanced penetration across the bacterial cell membrane, *in-vivo* stability, and specificity for intracellular targets (Christopher et al., 2021). Along with this, a higher concentration of antibiotics can be delivered inside the cells using these conjugates, which in turn limits the requirement of higher antibiotic dosage, thereby restricting its side effects (Kollef et al., 2011). In addition to this, pathogens that are multidrug-resistant pose the greatest threat to all antibiotics. The synergistic antibiotic release from NPs or several antibiotics conjugated or encapsulated on NPs provides further benefits in terms of bacterial killing effectiveness and speed.

The specific surface area of the antibiotic–NP system escalates as the size of the NPs decreases, allowing more antibiotic molecules to be functionalized onto the NP’s surface. The antibiotics can be linked physically or chemically to the NPs, without forming any core–corona structures. Hydrophobic, host–guest, avidin–biotin, and electrostatic interactions are all examples of physical interactions between antibiotics and NPs. In case of chemical interactions, different functional groups show conjugation with antibiotics by various means such as the following: amine-functionalized NP is conjugated with antibiotics that either contain a succinimidyl ester group or an isothiocyanate group, aldehyde-functionalized NP is conjugated with antibiotics that contain a hydrazide group, and active hydrogen-functionalized NP is conjugated with antibiotics that contain an amine group. AuNPs are conjugated with antibiotics that contain a sulfhydryl group, carboxyl acid-functionalized NPs are conjugated with antibiotics utilizing hexamethylene diisocyanate as a coupling agent and polyethylene glycol as a linker, alkyne-functionalized NPs are conjugated with antibiotics that contain an azide group, and tetrazine-functionalized NPs are conjugated with antibiotics that contain a trans-cyclooctene group (Jelinkova et al., 2019).

The antibiotic–nanomaterial system allows the desired amount of antibiotics to be delivered to the target sites with enhanced efficacy in cells. This can be accomplished *via* the following processes: i) antibiotic compounds can be hidden in antibiotic–nanomaterial conjugates during cellular penetration; ii) when antibiotics are administered at a high particle number concentration, bacteria are less likely to expel them through the efflux pumps; iii) when antibiotics are delivered in nanoscale form, they can be delivered more selectively and at a faster pace to the infection site. Due to differences in drug release kinetics, lower dosages of nanomaterial-conjugated multiple antibiotics are more efficacious than their molecular counterparts; iv) in infected cells, certain polymeric NPs have a higher biodegradability. Polymeric NPs can penetrate various membranes, break down inside the infected cell, and preferentially attach to contaminated components including the cytoplasm, macrophages, and phagolysosomes once delivered; v) inside cells, nanomaterials can preferentially accumulate and can capture microorganisms and prevent extracellular bacterial entrance into macrophages, reducing the severity of infections; vi) temperature, pH, light, ultrasound, magnetism, oxygen or carbon dioxide levels, ionic strength, and other stimuli-response characteristics of NP can be modified; and vii) multiple antibacterial pathways can be activated simultaneously by nanomaterials. A microbe’s ability to mutate is extremely unlikely. As a result, bacteria exposed to antibiotic–nanomaterial conjugates are less likely to acquire resistance (Mamun et al., 2021).

The antibiotic–nanomaterial conjugates work by combining the antibacterial property of the antibiotics with the advantages of the NPs providing a much better system for mitigating the development of drug-resistant pathogens. The mode of action for these antibiotic–nanomaterial conjugates for treating pathogens is very much similar to that of the NPs alone as discussed in the earlier sections. They mainly work by causing physical destruction of microbial cell structures, protein malfunction, membrane disintegration, ROS production and antioxidant depletion, and change of signal transduction through dephosphorylation of peptide substrates on tyrosine residues, resulting in signal transduction inhibition and bacterial growth suppression (Muzammil et al., 2018). Different examples of these antibiotic–nanomaterial conjugates used effectively for mitigating *E. coli* infection are given in **Table 5**.

**Table 5:** Various antibiotic–NP conjugates used for *Escherichia coli* mitigation.

S. no.	Nanoparticle	Size	Drug used	Mechanism of action	Reference
1.	AgNPs	5-40 nm	Chloramphenicol, ampicillin, kanamycin, and erythromycin	Bacterial cell wall interaction, interact with DNA, preventing it from unwinding	Fayaz et al. (2010)
2.	AgNPs	5-30 nm	Trimethoprim, vancomycin, imipenem, ciprofloxacin, and gentamicin	Cell wall synthesis disrupted and inhibited	Naqvi et al. (2013)
3.	AgNPs	–	Amoxicillin	Disintegration of negatively charged bacterial cell wall	Durán et al. (2010)
4.	AgNPs	28 nm	Colistin, penicillin G, gentamicin, and amoxicillin	Inhibit protein synthesis	Smekalova et al. (2016)
5.	AuNPs	33.9 ± 14 nm	Amoxicillin	Inhibit bacterial efflux pumps and synthesis of proteins	Kalita et al. (2016)
6.	AuNPs	7.8 ± 1.7 nm	Methylene blue	ROS and singlet oxygen generation	Perni et al. (2009)
7.	AuNPs	2.4 nm	Synthetic ligand	Upregulation of genes, inhibit bacterial efflux pumps and synthesis of proteins	Bresee et al. (2014)
8.	AuNPs	17.55 ± 2.95 nm	Cefotaxime	Disintegration of the bacterial cell wall and DNA damage	Shaikh et al. (2017)
9.	ZnO NPs	18-20 nm	Ciprofloxacin	Disruption of the cell membrane of bacteria	Patra et al. (2014)
10.	ZnO NPs	20-45 nm	Nitrofurantoin, amoxicillin, ciprofloxacin, and penicillin G	Disturb NorA protein pumping activity	Banoee et al. (2010)
11.	Penicillin-based carbon dots	4 nm	Penicillin	Light-activated ROS production	Luo et al. (2011)
12.	Fe_2_O_3_ NPs	10 nm	Gentamicin	Cellular DNA and RNA damage	Bhattacharya and Neogi (2017)
13.	SiNPs	50-80 nm	Tetracycline	Inhibit protein synthesis	Capeletti et al. (2014)
14.	SiNPs	72.4 ± 8.2 nm	Vancomycin and polymyxin B	Inhibit cell membrane synthesis	Gounani et al. (2019)
15.	PLGA-PLH-PEG (D,L-lactic-co-glycolic acid)-b-poly (L-histidine)-b-poly (ethylene glycol)	196 ± 7.8 nm	Vancomycin	Disrupt bacterial cell wall	Radovic-Moreno et al. (2012)
16.	Chitosan/Fe_3_O_4_/poly(ethylene glycol) PEG	30 nm	Gentamicin	Disrupt bacterial cell wall	Wang et al. (2018a)
17.	Carbon nanotubes	500 nm	Tetracycline	Inhibit bacterial efflux pump	Carver et al. (2020)
18.	CeO_2_ NPs	5-20 nm	Amoxicillin, cefotaxime, clavulanate, imipenem, and ampicillin	Increase permeability for antibiotics to diffuse passively through the outer cell membrane, increase oxidative stress	Bellio et al. (2018)
19.	CuO_2_ NPs	130 to 270 nm	Rifampicin	Disrupt bacterial cell wall	Woźniak-Budych et al. (2017)
20.	Poly(lactide-co-glycolide) (PLGA) and lignin-graft-PLGA (LNP)	111.8 ± 0.6 and 117.4 ± 0.9 nm, respectively	Enrofloxacin	ROS production	Paudel et al. (2021)

## 11 Antibiotic–NP conjugates at the industrial level or in clinical trials

Vaccinations were initially viewed as a promising preventative strategy and an inhibitory tool against microbial infections. Vaccines have the tendency to reduce the need for antibiotics and the illness symptoms (Baker et al., 2018). The development of a glycoconjugate and recombinant DNA technique makes the vaccination more effective against resistant pathogens such as *Pneumococcus*, *Haemophilus influenzae* type B, *Meningococcus*, group B *Streptococcus*, *Shigella*, and *E. coli* (Bilukha & Rosenstein, 2005; Delany et al., 2014). In recent years, the effective usage of nano-antibiotics in medicine has been noted. A proprietary formulation known as liposomal amikacin for inhalation (ARIKAYCE^®^, created by Insmed Incorporated) wraps aqueous amikacin in charged neutral liposomes made of dipalmitoyl phosphatidylcholine and cholesterol. The USFDA has authorized this nanoformulation for the treatment of patients with *Mycobacterium avium* complex lung illness, who have failed conventional therapy and have few or no other therapeutic options (Cipolla et al., 2016). Commercially accessible liposomal-encapsulated ciprofloxacin (Lipoquin™ or ARD-3100 and Pulmaquin™ or ARD-3150), both produced by Aradigm Corporation for non-cystic fibrosis bronchiectasis colonized with *P. aeruginosa*, is also available (Cipolla et al., 2016). Other than this, numerous nano-antibiotics, including mupirocin, gentamicin, polymyxin B, fluconazole, and others, are also being used in therapeutic settings. Prevnar is a pneumococcal vaccine created by Wyeth that contains saccharides of seven serotypes of the capsular antigens of *Streptococcus pneumoniae* coupled to mutant CRM197 diphtheria toxoid (Bricks & Berezin, 2006; Chakraborty et al., 2022). Other than these commercial nanoformulations, there are various ongoing studies for the development of nanoformulations having antibacterial and antimicrobial properties. Many studies have been reported for the development of antibiotic-conjugated nanomaterials that can be effectively used to mitigate bacterial pollution. For example, M. Martínez-Carmona et al. reported a novel antibiotic–nanomaterial conjugate based on mesoporous silica nanoparticles (MSNs) loaded with the antibiotic levofloxacin (LEVO) and coated with the lectin concanavalin A (ConA) (Martínez-Carmona et al., 2019). The stable MSNs having an average size of 150 nm were synthesized and loaded with LEVO. The ConA was then covalently attached to the MSN for easy penetration into the gram-negative bacteria biofilms, which in turn escalated the efficacy of LEVO to a great extent. These LEVO-loaded MSNs showed a significant increase in the antimicrobial property of LEVO against bacterial biofilms (Martínez-Carmona et al., 2019). In another study, A. Aguilar-Colomer et al. reported the antimicrobial activity of antibiotic cargo produced by MSNs against several bacterial strains, including *E. coli* and *S. aureus*, in detail (Aguilar-Colomer et al., 2021). The MSNs loaded with three different antibiotics, gentamicin, levofloxacin, and rifampin, separately were used to study their released dosages’ biological activity as well as their impact on bacterial biofilm. The study showed that MSNs loaded with gentamicin had an uninterrupted release kinetics but minimal antibiofilm impact. On the other hand, levofloxacin and rifampin revealed release profiles that included an early burst effect followed by a continuous release, with active dosages capable of reducing up to 99.9% bacterial biofilm and remaining active for 72 h. This study paves the way for the development of tailored MSN-based nanotherapies to treat chronic bone infection (Aguilar-Colomer et al., 2021). J. Aguilera-Correa et al. reported a nanomedicine capable of eradicating *E. coli*-related bone infections, engineered using MSNs as a platform (Aguilera-Correa et al., 2022). These MSNs were loaded with moxifloxacin and were further modified with Arabic gum and colistin. This antibiotic–Np system displayed a significant affinity for the *E. coli* biofilm matrix and a pronounced antibacterial effect because of the bactericidal impact of moxifloxacin and the disaggregating effect of colistin. The results showed that, *in vitro*, the antibiotic–nanomaterial system was able to mitigate an infection on a trabecular bone, and *in vivo*, it demonstrated substantial antibacterial activity against *E. coli*-induced osteomyelitis. The antibiotic–nanomaterial system was found to be preferably non-cytotoxic, having a non-significant effect on cell proliferation and a slight hepatotoxicity, possibly due to the characteristics of both antibiotics (Aguilera-Correa et al., 2022).

## 12 Econanotoxicity

Nanotoxicology is a distinct and relatively recent branch of study, as the behavior of materials at the nano level can differ from the bulk counterpart and enable unique properties for uptake or tissue interactions (Nel et al., 2006). Nanotoxicology of a particular material depends on several factors ranging from size, shape, constituent material, surface structure, surface charge, electronic structure, surface ligands, and solubility (Nel et al., 2006; Cassano et al., 2017). Human exposure to nanomaterials is nothing new, as natural nanomaterials are found mostly in the form of particulate matter in the air. The source of such nanomaterials are mostly volcanic, dust storms, cosmic, and some organisms (Kumar et al., 2021). Nanomaterials have several avenues from which they can enter the body, specifically *via* respiratory, dermal, and gastrointestinal routes. Since nanomaterials for the treatment of wastewater are mostly immobilized by the hydrosphere, only the dermal and gastrointestinal exposure routes are relevant. In a study, it was noted that neutral and positively charged latex nanoparticles in the 50-500-nm range could not penetrate the dermis, while negative ones could (Kumar et al., 2021). In another study, it was found that nanoparticles of 18 nm are absorbed by the GI walls of a murine model. Furthermore, it was reported that positively charged and smaller nanoparticles are more readily absorbed by the GI tract (Kumar et al., 2021). A study using ZnO and TiO_2_ nanoparticles on skin penetration showed low toxicity, but it varies with the chemical nature, shape, and an increase in size with some exceptions (Nohynek et al., 2007). Positively charged nanoparticles seem to pass through the cell membranes more easily than negatively charged or neutral nanoparticles. One explanation for this would be that positively charged nanoparticles aid in the adsorption of proteins forming a corona, which promotes opsonization (Garcés et al., 2021). Once the nanoparticles enter the skin, respiratory system, or GI tract, they can enter the lymph nodes and finally into the systemic circulation, where they can accumulate in various organs along with some particles being able to cross the blood–brain barrier. A study of various nanoparticles such as TiO_2_, ZnO, Fe_2_O_3_, Fe_3_O_4_, and MgO showed that the mode of toxicity included cardiorespiratory damage at the organ level as well as ROS and DNA damage at the cellular level (Wang and Tang, 2021). As previously stated, humans and other organisms have co-evolved with natural nanoparticles; however, their exposure to engineered nanoparticles is relatively very recent; hence, there is no evolutionary defense mechanism. Nanotoxicity in aquatic ecosystems is a major and recent concern, as increasing amounts of engineered nanoparticles are being used and finding their way into the hydrosphere. Nanoparticles with redox or dissolving properties have the potential to disrupt cellular membrane stability (Khezerlou et al., 2018; Chakraborty et al., 2022). If they enter the cell, they cause oxidative damage, so in general, they have a bacteriostatic effect and, in large enough concentrations, bactericidal effects. Algae is a cornerstone of aquatic ecosystems, acting as producers and supporting the food web. Nanoparticles have been demonstrated to adhere to algal cell walls and internalized by endocytosis and then move up the trophic level from there *via* ecological bioaccumulation. The main effects on algae on exposure to diverse engineered nanoparticles include membrane damage, reduced chlorophyll content, oxidative stress, mitochondrial depolarization, and altered gene expression. It was found that exposure of silver nanoparticles at 0.4 - 6.4 mg/L to *Dunaliella tertiolecta* and *Skeletonema costatum* reduced growth by half (Abbas et al., 2020). Engineered nanoparticles are also uptaken and accumulated in the food vacuole of protozoans *via* endocytosis and phagocytosis, and they can enter the food chain from there, leading to biomagnification. Exposure of engineered nanoparticles in plants can also lead to a host of reactions, which depends on their mode of uptake and translocation throughout different parts of the plant. Since sludge from wastewater treatment plants is also used as fertilizers, this is particularly relevant. Exposure of *Brassica oleracea* and *Lactuca sativa* to 10 and 250 mg/plant of CuO nanoparticles led to a reduction in plant growth and photosynthesis, whereas CuO nanoparticles when exposed to *Oryza sativa* at 50–1,000 mg/kg also led to negative effects to plant growth (Abbas et al., 2020). Exposure of *Ipomoea batatas* to CuO, CeO_2_, and ZnO nanoparticles at 100-1,000 mg/kg again led to a reduction in biomass and bioaccumulation (Abbas et al., 2020). Select metal oxide nanoparticles such as TiO_2_, Al_2_O_3_, and ZnO were detected in *Daphnia* gut kept in a nanosuspension for 48 h with TiO_2_, showing a rapid accumulation in 12 h and slow excretion and still remaining in the body in 72 h (Krysanov et al., 2010). TiO_2_ nanoparticles did not accumulate in the internal organs of rainbow trout; however, accumulation could be seen in the muscles and liver, when kept in a nanosolution (Krysanov et al., 2010). Tin was detected in the gills, gut, and spleen of guppy kept in a nanosolution of SnO_2_, whereas zebrafish kept in ZnO nanosolution did not accumulate the same in the gills, liver, brain, and kidney, but when exposed to CeO_2_ nanoparticles, they could be detected in the liver (Krysanov et al., 2010). The elimination and biodegradation of NPs through urine and biliary channel is very low. This allows the NPs to remain in the tissues for a longer period of time and accumulate in the liver, lung, spleen, bone marrow, colon, and lymphatic system; other than this, the harmful by-products from NPs may lyse RBCs, cause DNA damage, and obstruct blood coagulation pathways and become more hazardous (Lei et al., 2008; Ansari et al., 2014; Ivask et al., 2014; Kandi & Kandi, 2015; Lin, 2015; Zaidi et al., 2017). Therefore, the effects of exposure to engineered nanoparticles on the environment on a macro level can be broadly said to reduce productivity and cause bioaccumulation.

As previously mentioned, biota have no evolutionary defense against engineered nanoparticles; hence, the toxicity of NPs is a matter of recent concern; however, not all studies have been conducted at environmentally realistic concentrations. Also, the same properties that make nanoparticles useful and their small and active nature also contribute to their removal from the environment. The possible fate of such nanomaterials in wastewater treatment plants includes adsorption onto large debris which are separated by grit filters, aggregation and settling out in primary or secondary settling chambers, interaction and removal by interaction with organic and microbial matter, and oxidative removal in advanced disinfection processes (Brar et al., 2010). Removal by adsorption of organic or microbial matter, which is a major constituent of sludge, is an important fate of nanomaterials in wastewater treatment plants; hence, sludge resultant immediately after any nanomaterial application can be collected and dried, and after incineration, the nanomaterial is recycled. Natural aquatic environments are a very complex system of water, salts, humic acids, sediments, microorganisms, substrata, etc. Nanoparticles with high surface charge reduce homoagglomeration but promote hetero-agglomeration due to electrostatic attraction. Engineered nanoparticles collide in aquatic media and stick to solid surfaces which slows their transportation and results in sedimentation (Bathi et al., 2021). Despite this, active measures should be taken to reduce exposure and nanotoxicity to a bare minimum. Econanotoxicity can be minimized in broadly two ways which can be classified under quantitative and qualitative parameters. Qualitative parameters include the manufacture of nanoparticles which are least damaging to the environment by factoring in parameters which in general contribute to their nanotoxicity. Engineered nanoparticles should have the size and charge or combined surface charge density so that they are impermeable to the dermis as well as the GI tract. If possible, they should be biodegradable so that they do not cause nanobioaccumulation. Nanoparticles should be chemically stable, i.e., on unintentional release they should permanently stay as sediment and mineralize. They should not leach pollutants or transform into secondary pollutants. To achieve this, their constituents should be as beguine as possible. The morphology of nanoparticles should also be kept non-abrasive so as to not cause membrane damage if they are unintentionally released and encounter microbiota. Green nanosynthesis using phytoextracts can also be used to minimize ecological footprint (Sarkar et al., 2021). As previously mentioned, since nanoparticles contain a surface charge, they can be separated using an electrostatic screen. There is also the option of using magnetic-engineered nanoparticles which can be separated using an applied magnetic field (Nasrollahi, 2020). Quantitative methods include the minimization of the amount of total engineered nanomaterials used; in this regard, nanomaterials have an advantage because of their extremely high surface area to volume or mass ratio, which means that relatively small amounts of the material are required. Secondly, since nanoparticle-mediated activity depends on the surface properties and not on bulk volume, the engineered nanoparticles can be immobilized as a unilayer on a screen substratum using electroplating or sputtering, thereby using a little material as possible. Minimizing of nanomaterial used and retrieval is particularly useful in the case of antimicrobial nanomaterials, which by virtue of their function must be nanotoxic. As mentioned previously, another reason for the minimization of nanomaterial usage is the fact that nanoparticles adhere to the cell wall or membrane and destabilize it, making it more permeable. This can in turn promote the transfection of mobile genetic elements containing antibiotic resistance. As revealed in a previous study, exposure to sublethal concentrations of ZnO nanoparticles was shown to greatly enhance horizontal gene transfer from *E. coli*, hence highlighting the need for their judicial use (Wang et al., 2018b).

## 13 Conclusion

*Escherichia coli* is a very common gram-negative commensal which mostly lives in the large intestine of warm-blooded animals without causing any harm. However, under certain circumstances, it may acquire traits that make it pathogenic. The threat is compounded when one considers excreted *E. coli* in conjunction with antibiotic pollution which ends up in the same sink, that is wastewater treatment plants. Here, *E. coli* has been found to survive under directional evolutionary stress caused by these antibiotics by developing select pathogenic traits and, as a result, may develop and transfer antibiotic resistance *via* mobile genetic units which pose the risk of drug-resistant *E. coli* strains. These drug-resistant strains or “superbugs” can lead to diseases and epidemics which are resistant or untreatable by antibiotics conventionally available and stockpiled. Nanomaterials present a novel method in which this issue can be mitigated. Nanomaterials can be used preventatively as antibiotic conjugates, increasing their efficiency and reducing their need and thus discharge into the environment. They can also be used punitively as nanophotocatalytic agents either to lyse the cell wall using artificial or natural sunlight or to disintegrate antibiotic residues and mobile genetic elements in the environment. They can also be used as antimicrobial additives to decrease microbial population in the sink and, thus, the threat posed to humans *via* several avenues. However, it is important to remember that ecological threats posed by excessive discharge of engineered nanomaterials are probable but poorly understood; hence, nanomaterials should be used judiciously after considering all aspects of efficiency, human health, and environmental integrity.

## Author contributions

All authors listed have made a substantial, direct, and intellectual contribution to the work and approved it for publication.
